# Families’ Experiences of Continuous Glucose Monitoring in the Management of Congenital Hyperinsulinism: A Thematic Analysis

**DOI:** 10.3389/fendo.2022.894559

**Published:** 2022-07-19

**Authors:** Sameera Hannah Auckburally, Chris Worth, Maria Salomon-Estebanez, Jacqueline Nicholson, Simon Harper, Paul W. Nutter, Indraneel Banerjee

**Affiliations:** ^1^ Department of Paediatric Endocrinology, Royal Manchester Children’s Hospital, Manchester, United Kingdom; ^2^ Faculty of Health and Medicine, Lancaster University, Lancaster, United Kingdom; ^3^ Department of Computer Science, University of Manchester, Manchester, United Kingdom; ^4^ Paediatric Psychosocial Service, Royal Manchester Children’s Hospital, Manchester, United Kingdom; ^5^ Faculty of Biology, Medicine and Health, University of Manchester, Manchester, United Kingdom

**Keywords:** congenital hyperinsulinism, continuous glucose monitoring, thematic analysis, interviews, experiences

## Abstract

**Background and Aims:**

In patients with congenital hyperinsulinism (CHI), recurrent hypoglycaemia can lead to longstanding neurological impairments. At present, glycaemic monitoring is with intermittent fingerprick blood glucose testing but this lacks utility to identify patterns and misses hypoglycaemic episodes between tests. Although continuous glucose monitoring (CGM) is well established in type 1 diabetes, its use has only been described in small studies in patients with CHI. In such studies, medical perspectives have been provided without fully considering the views of families using CGM. In this qualitative study, we aimed to explore families’ experiences of using CGM in order to inform future clinical strategies for the management of CHI.

**Methods:**

Ten patients with CHI in a specialist centre used CGM for twelve weeks. All were invited to participate. Semi-structured interviews were conducted with nine families in whom patient ages ranged between two and seventeen years. Transcripts of the audio-recorded interviews were analysed using an inductive thematic analysis method.

**Results:**

Analysis revealed five core themes: CGM’s function as an educational tool; behavioural changes; positive experiences; negative experiences; and design improvements. Close monitoring and retrospective analysis of glucose trends allowed for enhanced understanding of factors that influenced glucose levels at various times of the day. Parents noted more hypoglycaemic episodes than previously encountered through fingerprick tests; this new knowledge prompted modification of daily routines to prevent and improve the management of hypoglycaemia. CGM use was viewed favourably as offering parental reassurance, reduced fingerprick tests and predictive warnings. However, families also reported unfavourable aspects of alarms and questionable accuracy at low glucose levels. Adolescents were frustrated by the short proximity range for data transmission resulting in the need to always carry a separate receiver. Overall, families were positive about the use of CGM but expected application to be tailored to their child’s medical condition.

**Conclusions:**

Patients and families with CHI using CGM noticed trends in glucose levels which motivated behavioural changes to reduce hypoglycaemia with advantages outweighing disadvantages. They expected CHI-specific modifications to enhance utility. Future design of CGM should incorporate end users’ opinions and experiences for optimal glycaemic monitoring of CHI.

## Introduction

Congenital hyperinsulinism (CHI) is a disorder characterised by severe hypoglycaemia due to inappropriate secretion of insulin by the pancreatic β-cells (1). Despite CHI being a rare disorder with an estimated incidence of 1:28,389 in the UK, it is the most common cause of persistent hypoglycaemia in children (2, 3).

In addition to causing hypoglycaemia, excessive and dysregulated insulin secretion suppresses the production of ketones, which normally act as an important alternative fuel to preserve neuronal function when there is insufficient glucose (4). CHI is therefore well-recognised for its association with poor neurodevelopmental outcomes in patients, with 15% - 48% of children with CHI having long-term neurodevelopmental impairment at follow-up (4–7). Prompt detection and treatment of hypoglycaemia in CHI is therefore vital. Standard clinical practice for the monitoring of glucose in CHI is with regular fingerprick blood glucose testing using a point-of-care device or a home glucometer, whilst management includes the optimisation of feeds, medications such as diazoxide and octreotide, and pancreatectomy dependent on the type of CHI (8).

Advancements in technology have resulted in the increasingly widespread use of continuous glucose monitoring (CGM) rather than fingerprick blood glucose monitoring in patients with type 1 diabetes mellitus (9). The minimally invasive CGM device is attached to the skin, detects changes in interstitial glucose levels and displays the readings to the user every five minutes via a hand-held receiver or a mobile phone (10). Frequent glucose monitoring is a cornerstone of intensive CHI management and CGM provides an attractive alternative to intermittent fingerprick testing, which has too low a granularity to offer trend information and can miss hypoglycaemic episodes in between measurements.

Although there is heightened interest about the use of CGM in patients with CHI, the clinical utility has not been explored carefully (11). There is growing interest in the application of CGM to improve glycaemic control in neonates with CHI (12, 13). Win et al. reported that CGM showed rapid fluctuations in glucose levels in fourteen neonates with CHI alongside persistent hypoglycaemia, reflecting the high risk of undetected hypoglycaemic episodes when managed on intermittent fingerprick glucose tests (14). Rayannavar *et al*’s observational study demonstrated a high false positive rate for hypoglycaemia readings for children with CHI over a two-week period; the authors determined that CGM should be used as an adjunct to glucose monitoring rather than a sole monitoring device due to its suboptimal accuracy (15). More recently, Worth et al. conducted an exploratory study in which CGM was used to collect detailed glycaemic data over a period of four to ten days in twenty-three patients with CHI and found that there was an increased risk of hypoglycaemia in the early hours of the morning (16).

The HI Global Registry, a patient-powered CHI registry, found that 49% of parents of children under five reported the management of CHI to be ‘demanding’ (17). A recent review considering the unmet needs of patients and families with CHI suggested the wider application of CGM, while recognising shortcomings in its present use (11). While CGM has the potential to improve glycaemic monitoring and hence outcomes in CHI, it is vital that end users’ opinions on using the device are gathered before broader implementation. By way of a questionnaire, Vijayanand et al. sought to evaluate parents’ experiences of CGM; the majority preferred using CGM to a fingerprick glucometer, although seven out of the eleven parents felt that it was not accurate all the time (18). However, deeper analysis of patients’ and families’ experiences through interview was not available. In our study, we aimed to gain a richer understanding of the experiences of families using CGM; we conducted the first qualitative study employing thematic analysis of semi-structured interviews with adolescents with CHI and parents of young children with CHI.

## Methods

We undertook a qualitative study to perform an in-depth analysis of families’ experiences of CGM use in a small group (n=9) of CHI patients. As little is known about the experiences of CGM in families with CHI, a rare disease of hypoglycaemia, qualitative methods are ideal for investigating the subject in a small targeted population in contrast to structured questionnaires in a larger group (19). They allow for participants to freely disclose their thoughts and experiences without constraint, providing a unique depth of understanding that cannot be gained from a closed question survey (20). Futhermore, qualitative methods and analysis enable open representation of user perceived concepts and themes reducing prior prejudice and investigator bias from influencing the results.

This qualitative study was the second phase of a related study in which patients with CHI had used CGM (Dexcom G6) for twelve weeks and received expert review of glucose profiles at weeks eight and twelve without pharmacological intervention (21). The Dexcom G6 used in the study employed a separate hand-held receiver. CGM glucose was reported in mmol/L; as per UK consensus, hypoglycaemia was defined as less than 3.5mmol/l (63mg/dL) (2). If CGM reported a glucose level of less than 3.5 mmol/l, families were instructed to also check the glucose level with a fingerprick blood glucose test and treat hypoglycaemia if confirmed.

During the first four weeks, families were blinded to the glucose readings, which are usually displayed in real time by CGM. They were then able to use CGM unblinded, with readings available for four weeks, before a review of the glucose trends during this time period was conducted with a research clinician. For the final four weeks of the study, the device was blinded once more and followed by a final review of glucose profile. For inclusion in the study, patients with CHI were approached through the Northern Congenital Hyperinsulinism Service (NORCHI), Manchester, United Kingdom. Patients were eligible for inclusion to the CGM study if they were under the age of eighteen years and receiving medication for treatment of confirmed CHI.

For this qualitative study, inclusion criteria included parents/guardians of children with CHI, adolescents with CHI (defined as greater than twelve years of age) and the use of CGM for at least six weeks during the study period (including four weeks of unblinded CGM). All families of participants of the initial study were approached to be included in the qualitative phase of the study. All ten families initially consented to participate in the study. However, one family did not maintain contact thus preventing them from inclusion in the interviews. Two of the five adolescents did not participate in the interview alongside their parent(s) after having previously consented to participation; this was due to fatigue at the time of interview for Patient 3; and the mother of Patient 5 only being available for interview during the day, whilst her daughter was at school.

The protocol, consent forms and interview topic guide were approved by the ethics committee of the University of Manchester and the Health Research Authority of the National Health Service (REC reference 07/H1010/88). Adolescents and parents of the younger children gave written informed consent. Further verbal consent was obtained at routine research follow-up clinic appointments prior to organising interviews. Incentives were not provided for participation.

Semi-structured interviews with parents and adolescents were conducted in December 2021 via videoconferencing platforms to explore families’ experiences of CGM use. At the time of interview, all families had used CGM for twelve weeks during the study and had continued to use unblinded CGM for a further four weeks. The semi-structured approach was selected as it permitted the flexibility for participants to speak about the issues they perceived to be most important, and for those to be explored, whilst the topic guide helped to ensure data collection remained relevant to the study aim. The **The Appendix within the Supplementary Material** includes the interview topic guide which consisted of prompts and questions on families’ opinions and experiences of CGM and the perceived benefits and challenges of using CGM. The interview guide was developed through consensus with researchers, clinicians and psychologists with expertise in CHI.

Braun and Clarke’s approach to thematic analysis was chosen as the mode of analysis as it allowed a pragmatic approach to analyse participants’ lived experiences, behaviours, and perspectives (22). Its flexibility also allows for use on small datasets, which is especially important given CHI is a rare condition. Thematic analysis was favoured over interpretative phenomenological analysis, which can also be conducted on small homogenous samples, as it places greater emphasis on patterns across participants whilst the latter phenomenological approach notes patterns but focuses on how each unique individual makes sense of events (23).

In terms of reflexivity, interviews were conducted by a clinical research paediatrician who was not involved in the first phase of the CGM research study and did not have prior information about the patients or families. Importantly, the families understood that reporting on their experiences would not affect their potential future supplies of CGM equipment or clinical care. Members of the same family were interviewed together and each interview lasted between twenty and thirty minutes. Respondent validation, whereby participants confirmed accuracy of the information they had provided, was conducted throughout the interview process.

Interviews were transcribed verbatim and subsequently checked for accuracy against the audio recordings. This stage, along with repeated reading of the transcripts and noting early impressions, allowed for further familiarisation with the data. Personal identifiers were removed in the transcription process.

As there was little predetermined knowledge about CGM experiences in CHI, a predominantly inductive approach was used to code the data. Hence, research findings were derived from the data rather than using a pre-defined coding framework. It was not deemed appropriate to use multiple coders in this thematic analysis approach; inter-coder reliability merely shows that researchers have been trained to interpret data in similar ways (24, 25). Qualitative data analysis software (NVivo) was used to facilitate the application of initial codes to the entire dataset. Multiple codes were then combined to create themes, which captured common, recurring patterns across the data that described and explained participants’ experiences. Prior to defining and naming of the themes, they were refined by reviewing all collated extracts for each theme to ensure there was sufficient supporting data.

## Results

Nine families were included in the study of which there were five parents of younger children, five parents of adolescents and three adolescents. For each patient with CHI, at least one parent or patient was involved in the interview as described in **Table 1**. The demographics of patients, alongside CHI medications and the time since diagnosis are also presented in **Table 1**.

**Table 1:** Interview participants and demographics of patients with CHI.

Interview Participant	Patient	Age at Time of Interview/years	Gender	Time since diagnosis of CHI/years	Genetics	Medications
**Participant 1 [P1]**-Father of Patient 1	Patient 1	3.1	Male	3.1	Homozygous ABCC8 mutation	Subcutaneous injections of octreotide three times daily
**Participant 2 [P2]**-Patient 2	Patient 2	14.5	Female	14.5	Paternally inherited KCNJ11 mutation	Oral diazoxide twice daily
**Participant 3 [P3]** -Mother of Patient 2						
**Participant 4 [P4]** -Mother of Patient 3	Patient 3	12.3	Male	11.9	No genetic cause identified	Oral diazoxide twice daily
**Participant 5 [P5]** -Mother of Patient 4	Patient 4	5.4	Male	5.4	Maternally inherited ABCC8 mutation	Oral diazoxide three times daily
**Participant 6 [P6]** -Mother of Patient 5	Patient 5	13.3	Female	13.0	HADH mutation	Oral diazoxide twice daily
**Participant 7 [P7]** -Patient 6	Patient 6	17.7	Male	7.4	GCK mutation	Oral diazoxide twice daily
**Participant 8 [P8]** -Mother of Patient 6						
**Participant 9 [P9]** -Mother of Patient 7	Patient 7	3.2	Female	3.0	No genetic cause identified	Oral diazoxide three times daily, chlorothiazide twice daily, cornstarch
**Participant 10 [P10]**-Mother of Patient 8	Patient 8	2.1	Male	2.1	HNF4A mutation	Oral diazoxide three times daily
**Participant 11 [P11]** -Father of Patient 8						
**Participant 12 [P12]** -Patient 9	Patient 9	17.3	Male	17.1	GLUD1 mutation	Oral diazoxide three times daily
**Participant 13 [P13]**-Mother of Patient 9						

The results are presented as five major themes that were derived from the data:

1. Positive Experiences
2. Educational Tool
3. Behavioural Change
4. Negative Experiences
5. Design Improvements.

A rich and detailed analysis of the themes are accompanied by illustrative quotations to ensure robustness, whilst **Table 2** provides a summary of the themes in families’ experiences of CGM in CHI.

**Table 2:** Description of 5 major themes and subthemes in families’ experiences of CGM use in CHI.

Theme	Positive Experiences	Educational Tool	Behavioural Change	Negative Experiences	Design Improvements
**Theme Description**	Factors regarded as positive/helpful by participants	Learning from CGM to improve management	Changes to routine due to CGM	Factors regarded as negative by participants	Refinements to design of CGM

**Subthemes**	Reassurance	New knowledge of glucose trends	Timing of meals changed	Alarms	Increase receiver range
	Less stressful management	More hypoglycaemia than previously thought	Ensured medications given on time	The need to carry receiver due to range	Incorporate wearable receiver

	Reduced fingerprick tests	Heightened awareness of hypoglycaemic times of the day	Improved family dynamics	Accuracy	Sensor size

	Glucose trend predictions	Reflection on reasons for hypoglycaemias	Adolescents taking increased responsibility for own condition	Sensor insertion	Tailor CGM for those with CHI e.g. improve accuracy at lower glucose levels
	Objective evidence of low glucose	Adhesive problems			

	Optimisation of blood glucose control

### Positive Experiences

Whilst families had become accustomed to living with CHI, their day-to-day life was felt to be less stressful with CGM as they were reassured about normoglycaemia, especially at night-time. *“But again, it’s just peace of mind for parents that - just to see what’s happening. Especially at night - if it works at night when she’s poorly then I don’t have to prick her as it might wake her up. So it’s really good at night.” – P9.*

Managing the condition was also perceived to be simpler with CGM enabling families to have more time to focus on other matters. Older siblings were also able to gain a sense of responsibility by helping out. *“When you’ve got a lot of other medical issues going on, it’s just one thing that makes life a lot easier. Life’s quite hectic. So we’ve got that one little thing that you ain’t got to do which is like checking his blood sugars every time we eat.”- P11. “Even our teenage children - they can be aware of it as well, because they haven’t got to start messing with the pins and whatever. They can just look on the monitor. Gives us a little bit, not a lot, but a bit more leeway of doing other things in the house.” – P10.*

A significant positive outcome through the use of CGM was the reduced number of fingerprick tests required: *“Also I like the fact I’m not having to check his bloods myself as much so that saves his little fingers.”- P5*. Parents of younger children felt that day-to-day life for their children was less disrupted, especially at nursery and when playing outside, which would often require finding a space to remove clothing to do fingerprick tests. One parent [P6] discussed the environment: they perceived CGM to be more environmentally friendly than fingerprick monitoring techniques, which resulted in a perceived increase of non-recyclable wastage of testing strips and needles.

Parents of adolescents liked that CGM assisted them with objective evidence of hypoglycaemic episodes that could not be ignored continually by their children. Previously, as they were asymptomatic, the adolescent would often report that a fingerprick test was not required. *“We were saying to him, well I know for a fact that your sugars are 2.5, you need to have something to eat” – P4 “But now we both can see that, okay, it’s low, and she’ll read it’s low, rather than us arguing.” – P3.*

CGM’s feature of predicting future glucose values was found to be particularly useful to prevent hypoglycaemia: *“because it does kind of alert you if my son’s about to have a low and then I can act on that “- P5* Through predictive warnings and optimisation of mealtimes and medication timings, CGM allowed for general and persistent improvement in blood glucose control compared to management pre-CGM. *“Mainly, in my opinion, it [CGM] has helped [patient’s name] not get any low sugars and to contain his sugar levels, which, obviously, low sugar levels are not good for you anyway. So, in our opinion, it’s helped us not get any low sugars.” – P1.*

### Educational Tool

CGM was perceived to be an enlightening educational tool; the technology allowed families to obtain new knowledge about glucose trends specific to their child: *“It takes about an hour, just over an hour, for his sugar levels to go up. We used to be under the impression that the [octreotide] injection takes fifteen minutes and that could tell us but that wasn’t the case so we realised to pay a lot more attention between feeds” – P1.* Most young patients with CHI are unable to verbalise symptoms of hypoglycaemia and many develop relative hypoglycaemia unawareness through recurrence (26); for these reasons, CGM was described as a *“lifechanger”* as it drew attention to low glucose levels when there were no demonstrable signs of hypoglycaemia. *“If it wasn’t for that machine, I wouldn’t even know, because my son doesn’t even display any symptoms” – P5.*

Parents felt that they had been managing the condition appropriately prior to study participation but were surprised by the unexpected number of hypoglycaemias highlighted by CGM, especially in between the times of their usual fingerprick tests. Having gained the new information from CGM about recurrent hypoglycaemia, families had heightened awareness of low glucose levels at certain times of the day: *“It kind of made you more aware that when you had access to it, you knew that it was going to dip at a certain point and you thought – ‘well, he’s just played football for an hour and a half, he’s going to need something’ “ – P4.* This allowed parents to reflect and analyse the possible reasons for hypoglycaemia at specific times, prompting preventative action to achieve normoglycaemia.

### Behavioural Change

Long-term behavioural change due to CGM was noted in all but one of the families, especially with regards to mealtimes. *“So we would make sure that he had something a bit more sugary in the evening or have a late dinner, just to make up for those late hours in the morning where he’s getting those low sugars” – P1*. *“It showed some certain times I was getting a lower, like, say on a Friday morning, because I start late, I don’t get out of bed until later on so I start-my blood was dropping so I then did end up making a slight change to my diet by eating, by making sure I definitely ate the night before and waking up slightly earlier.” – P7.*

The timing of medications was not generally changed by families, but there was increased appreciation for ensuring medications were not missed and given on time as it was noted that glucose levels gradually decreased as the time for medication approached. *“I give her medications earlier most of the time. Her normal dose should be at midnight, but I’m really tired most of the night. I can’t stay up until then. Normally when I sleep before midnight, chances are, I would miss her midnight dose. And then she would wake up with a low in the morning.”* – *P3*. CGM was also thought to potentially influence dose adjustments as thorough review of glucose trends was undertaken by families and clinicians. *“But there is talks of hopefully dropping the daytime dose of diazoxide. Maybe we wouldn’t have been able to do that if it weren’t for the Dexcom. We’ve been able to monitor it more closely. But because we’ve had a couple of lows, they’ve told us to hold back on it but hopefully soon.” - P10.*

Pre-CGM, parents described having to persistently remind their older children to check their glucose levels with fingerprick tests and manage their condition effectively. Because of CGM, family dynamics were reported to have changed for the better: *“We are more calm as well, me and her, we don’t argue more because our arguments always stem from her testing her blood sugars. So it kind of reduces that as well. It makes it more peaceful.” – P3.* CGM use also allowed for adolescents to gain more independence and responsibility as they developed further understanding of their own condition through monitoring of glucose trends, rather than performing fingerprick tests purely because they were told to do so. *“So she has got her snacks with her - if she’s gone to another lesson – she knows, right, my sugar’s this and, I think, by the time I get to another lesson, it might go down so she’s advanced, she’s had something to eat. So then it stays really good.” – P6.*

### Negative Experiences

Although feedback was largely positive, barriers to CGM use were described, such as disruption from alarms, accuracy, sensor insertion and problems with the receiver range.

All participants independently raised the issue of alarms. When using CGM initially, the alarms due to hypoglycaemia caused panic in the parents of younger children. With increased familiarity with CGM, parents would simply check their child’s glucose level with a fingerprick test and act accordingly. However, some families expressed frustration at the constant disruptions, especially at night and at school, resulting in an element of alarm fatigue. *“and then when they are actually low, it just-it just went crazy to be honest. We were kind of thinking, to the point where we had to actually turn it off so we could sleep.”- P3.* For one adolescent, it seemed CGM audibly distinguished her as different from the rest of her class. She wished to keep her condition private and the alarms accompanying CGM were not discreet in that regard. However, the other adolescents did not acknowledge similar problems at school.

An alarm was also triggered when the receiver was out of range of the sensor, which would occur at a distance of greater than six metres. Adolescents strongly disliked having to always carry the receiver with them and would often forget the receiver, resulting in further frustration from an activated alarm. “*Well, ‘cause it’s just annoying having to carry, like, a monitor in my pocket, where I have to know where it is” – P12. “Or sometimes she’ll go to the toilet and she’ll forget to take it with her and it’ll beep. And she’ll be like ‘Oh my god, I’m in the toilet!’ – P3.*

Families questioned the accuracy of the CGM readings. A low glucose reading of less than 3.5 mmol/l would trigger an alarm as advised and set by the clinical team. However, a check fingerprick test would typically demonstrate a higher blood glucose level than the CGM value. *“There was a few times where the machine was going low, but actually when we tested it, for [patient’s name] it was fine. So yeah, it was a bit of a tricky..like ooh what do you believe?” – P12.* Participants believed the inaccuracy at low glucose levels was the reason for many of the unnecessary alarms during the study period: “*It seems to do that quite a lot where the machine is just beeping, beeping, beeping – you know his glucose levels are 2.4. It’s like in my mind that doesn’t even make sense because actually well he’s on a nighttime feed, he’s getting milk, so how can it be?” – P5. “It was, giving us, like, these warnings to say that her blood sugars were low. It wasn’t. It was actually not a lot of times. And it kind of was a bit of a nuisance for a while, because it would beep, just unnecessarily” – P3.*

A challenging aspect for the majority of parents with young children was changing the sensor, which took place every eight to ten days. They viewed the sensor changes to be somewhat uncomfortable and frightening for children. *“I would love to have it [CGM] permanently for my son even though he does have that episode of going crazy when I’m changing it and putting it on. It’s quite hard for me as well, ‘cause he is really, gets himself really worked up. But I’m okay with him getting worked up for those 5 minutes, because of what the machine provides.”- P5.*

Parents also reported problems related to the adhesive used to attach the sensor to the body: *“It’s really difficult to take off even with the … I bought this ‘Zoff’ – everybody has tried to use that. It helps but it’s still very sticky, which obviously keeps it in place. It’s just when we’re trying to remove it, it’s not too pleasant.” – P9*. This was generally apparent in younger children, but the opposite was noted with one adolescent: *“I do think, however, the sticky plaster around it is not strong enough. Especially for [patient’s name], ‘cause he’s hairy.”- P13.*

The blinded aspect of the study was understandably not appreciated; adolescents felt it was pointless to carry a device that did not supply immediate glucose readings and parents felt increased anxiety as they had started to rely on the unblinded CGM readings. *“So, it’s almost made you a little bit on edge because you’re thinking ‘Do I test his sugars? I know he said he’s alright and he’s not had anything to eat, but this time last week when we could see the results, it was saying this and so is it going to be like that? Do I need to check him? Does he feel alright? Does he not feel alright?’ “ – P4.* However, there was some subtle acknowledgement that families can become fixated on the readings: one parent of an adolescent preferred the CGM readings being blinded to her as she *“wasn’t looking at it all the time. You do become a little bit obsessed with it, especially when it’s unblinded, because you’re just constantly looking at the readings.” – P13.*

The families were able to use the CGM device as part of a study and therefore did not require self-payment for device components or consumables. However, they acknowledged the high cost of the supplies would potentially prevent continued usage of the technology. *“To fund it yourself, it’s a lot of money. I think it’s about £150-200 a month, which is a lot of money.” – P5. “We’ve found it quite more easy but cost-wise it’s quite expensive as well.” – P6.*

### Design Improvements

The families described some improvements they felt could be made before potential widespread usage of the technology for those with CHI.

A short proximity range for data transmission was cited as a problem. This would be aggravated during routine daily activities such as during sport or when the receiver was accidentally forgotten. The range was identified by adolescents and their parents as one of the main improvements they would like to occur to the existing device. *“Or if he’s playing football obviously, we leave it at the halfway line in his bag. Then obviously it doesn’t always pick up because you’re not within that distance that it should be” – P12. “So he could give it to one of his coaches during a rugby game or rugby training, because he can’t physically have it on him when he’s tackling. So the range if it was slightly longer, it would be more beneficial for him.” - P8.* Parents of younger children bypassed the issue by using a small bag strapped across the child to hold the receiver when the child was at nursery or outside *“yeah, you have to carry it around, that’s quite..yeah..not annoying, but, like it could be a bit better. [Patient’s name] wears a little pouch around her to carry it around.” – P9.*

An alternative design improvement to the issue of receiver range was for the sensor to transmit signals to a mobile phone or a wearable receiver, such as a watch: “*that monitor should be in to like a watch so that they can just wear it, you know, round their wrist. So that’s 24 hours with them, the whole day. And they don’t take it off.”- P6.* It should be noted that this is available for older children and adults, but a separate receiver was used for the first phase of the study.

Families expected CGM interpretation and use to be tailored for those with CHI. They appreciated that CGM had been initially designed for those with type 1 diabetes, however they thought increased utility could be gained from improved accuracy at lower glucose levels and refined predictive warnings based on glucose trends from those with CHI: *“Sometimes it’s expecting what it’s going to be a blood sugar and obviously they base it on diabetic people. I think they, how the sugar levels go up and down is different than [patient’s name] so maybe diabetics shoots very quickly, [patient’s name] less so” – P9.*

Participants thought that a smaller sensor could improve the level of comfort. As the CGM sensor was attached to the body (often on the abdominal wall), the sensor could sometimes disrupt sleep and be obstructive for fastening trousers. *“The monitor that we put on the stomach, the CGM thing. I think we wanted it to be a bit smaller. It was quite big. I think if it was a bit more smaller, they would find it a bit comfortable.”– P6.*

## Discussion

This study is the first qualitative exploration of experiences of CGM amongst families of patients with CHI. The strengths in this study lie in gathering rich data on the experiences of both adolescents and parents of younger children, with matters examined in depth within semi-structured interviews. Furthermore, the analysis was conducted by an investigator without previous involvement in CGM studies in CHI or prior information about the patients, allowing for an inductive coding approach limiting researcher bias. We have observed emergent themes in this study, such as CGM being a catalyst for behavioural change and end users’ design improvements of the device, that have not been reported before. This study highlights the importance of incorporating end-user experience in the clinical application of medical devices. While innovations in healthcare and adoption of new technologies such as CGM are welcomed in CHI (11), it should not be assumed that end users will invariably favour CGM over fingerprick tests for glucose monitoring. It is important that user experience is factored in the clinical decision for use of CGM in patients with CHI.

All parents wished to continue to use CGM in the future with the view that the positive aspects, such as continued reassurance, predictive warnings, and less demanding glycaemic management outweighed the disadvantages. Adolescents, however, were more reluctant to carry on using the same version of the device due to problems with range, frustration with alarms and the need to carry a separate receiver.

Families noted that close monitoring and retrospective analysis of glucose trends enhanced their understanding of factors that influence glucose levels at different times of the day. Although parents were surprised that CGM revealed more hypoglycaemic episodes than previously encountered through intermittent fingerprick tests, the new knowledge obtained from CGM allowed for modification of routines to improve management of CHI.

Refinements to the design of CGM were discussed by participants; interestingly, it was clear to families that the system had not been designed for patients with CHI. In keeping with clinician perspectives (27), parents and patients with CHI commented on the need for future work on glucose forecasting algorithms to improve hypoglycaemia accuracy and predictions in CHI.

Participants’ experiences with poor accuracy is in keeping with previous studies: flash glucose monitoring systems were found to overestimate glucose levels compared to fingerprick tests (28), whilst CGM measurements, on average, were lower than fingerprick glucose measurements (15). Furthermore, Worth et al. reported a hypoglycaemia sensitivity of 44% with the Dexcom G6 device [unpublished data, in submission]. In response to questionnaires, most of the parents within Vijayanand et al’s study reported better sleep (18). In our study, whilst there was increased parental reassurance of normoglycaemia during the night, participants’ sleep was often disrupted due to alarms, especially on initial use of CGM.

Whilst parents reported on their experiences and on behalf of their young children, full exploration of children’s views was not grasped in this study. This was also the case for two out of the five adolescents who were either unavailable or did not wish to participate in the discussion. The adolescents that did participate were interviewed alongside a parent, which allowed for parental clarification of adolescents’ points. However, although every effort was made by the interviewing researcher to fully explore the young people’s views, the presence of parents may have unintentionally limited the adolescents’ contributions.

One family that withdrew from the previous phase of the study initially agreed to participate in the qualitative study but was subsequently lost to follow up arrangements. Exclusion of this patient may have introduced an element of positive bias into the remainder analysis. Future efforts should actively seek out those who did not find CGM useful. Interestingly, whilst the other nine out of ten families completed all twelve weeks of the CGM study; at the time of writing, three further patients have since withdrawn from follow-up provision of CGM now that regular expert review of glucose profiles has ceased, suggesting that unsupported CGM at home is not universally popular amongst those with CHI and participating in the parent study may have unintentionally influenced the findings of the analysis.

A nationwide survey is being developed to be distributed to patients with CHI in the UK to gather the views of all families using CGM. This process of methodological triangulation will aim to establish further validity of the findings from the thematic analysis.

In conclusion, the family experience for the use of CGM in CHI was generally positive. CGM allowed families to learn from glucose trends, prompting the prevention of hypoglycaemic episodes with simple routine changes. Whilst CGM increased reassurance and patients had fewer fingerprick tests, participants disliked the receiver proximity range and alarms. Attention to CHI-specific modifications for CGM, such as improved hypoglycaemic accuracy, is needed to enhance the end user experience for this often underserved patient group.

## Data Availability Statement

The raw data supporting the conclusions of this article will be made available by the authors, without undue reservation.

## Ethics Statement

The studies involving human participants were reviewed and approved by Ethics Committee of the University of Manchester and the Health Research Authority of the National Health Service (REC reference 07/H1010/88). Written informed consent to participate in this study was provided by the participants’ legal guardian/next of kin. Written informed consent was obtained from the minor(s)’ legal guardian/next of kin for the publication of any potentially identifiable images or data included in this article.

## Author Contributions

SA, CW, and IB contributed to conception and design of the study. SA conducted the interviews and analysis and wrote the first draft of the manuscript. All authors contributed to manuscript revision, read, and approved the submitted version.

## Funding

CGM devices were funded by a Momentum fund grant from Health Innovation Manchester - Momentum Fund Award [CGM in CHI (Cardio)].

## Conflict of Interest

The authors declare that the research was conducted in the absence of any commercial or financial relationships that could be construed as a potential conflict of interest.

## Publisher’s Note

All claims expressed in this article are solely those of the authors and do not necessarily represent those of their affiliated organizations, or those of the publisher, the editors and the reviewers. Any product that may be evaluated in this article, or claim that may be made by its manufacturer, is not guaranteed or endorsed by the publisher.
