# Supplementary material for: Families’ Experiences of Continuous Glucose Monitoring in the Management of Congenital Hyperinsulinism: A Thematic Analysis
Source: Front Endocrinol (Lausanne). 2022 Jul 19;13:894559. doi: 10.3389/fendo.2022.894559 (PMC9343578; doi:10.3389/fendo.2022.894559)
Supplement: Supplementary file 1 [file DataSheet_1.pdf]

northern congenital hyperinsulinism service

The University of Manchester

Appendix: Topic Guide for Interviewer

Note to interviewer: This document is a guide to the main themes and issues to be covered. Questions can be modified and followed up in more detail as appropriate.

Introduction

Initial thank you to individual/family for agreeing to talk to us.
Introduction of yourself and your role as member of research team.

Explain the reasons for the interview – to find out more about their experiences of continuous glucose monitoring (CGM). Explain that their experiences will help us to identify if CGM could potentially be used as part of standard management in patients with congenital hyperinsulinism (CHI).
Outline that the interview will last a maximum of 30 minutes.
Explain that the person is free to ask questions, decline to answer specific questions and can stop the interview at any time if they wish to.
Findings will be written up and published in a scientific journal or presented at a conference.
The interview will be audio recorded as it helps us to capture exactly what has been said; quotes will be used in the publication, but will be anonymised. Explain that they can check their individual transcript afterwards for accuracy.

Ask if there are any questions before commencing with the interview.
Ask them to verbally confirm that they understand the objectives and confidentiality of the research and that they are happy to participate.

Experiences

Could you tell me what you thought of the CGM device.
Describe your experience of using CGM.

Prompts if needed: What did you like about CGM? What did you find helpful about CGM?
Describe any negative aspects of CGM that you encountered.
What did you find challenging about using the CGM?
In what ways did the CGM impact on your daily life?
Tell me about if the CGM changed your routine?
How did your behaviour change (if at all) after going through the data in clinic?

Access to Data

What effect did seeing the real time glucose readings have on you?
How did it feel to go from having access to real time glucose readings to not having access at all for the last four weeks?

Expectations and Future

How did using the CGM compare to how you expected it to be?
Do you think you would use CGM again in the future if it was available? Why or why not?

Ending the Interview

Thank participant for their time.
Ask if there are any questions.
